# Time-series maps of aboveground carbon stocks in the forests of central Sumatra

**DOI:** 10.1186/s13021-015-0034-5

**Published:** 2015-09-17

**Authors:** Rajesh Bahadur Thapa, Takeshi Motohka, Manabu Watanabe, Masanobu Shimada

**Affiliations:** Earth Observation Research Center, Japan Aerospace Exploration Agency (JAXA), 2-1-1 Sengen, Tsukuba, Ibaraki 305-8505 Japan

**Keywords:** Forest carbon, Deforestation, PALSAR, AFCS, Aboveground biomass, REDD+, Riau

## Abstract

**Background:**

Efforts to reduce emissions from deforestation and forest degradation in tropical Asia require accurate high-resolution mapping of forest carbon stocks and predictions of their likely future variation. Here we combine radar and LiDAR with field measurements to create a high-resolution aboveground forest carbon stock (AFCS) map and use spatial modeling to present probable future AFCS changes for the Riau province of central Sumatra.

**Results:**

Our map provides spatially explicit estimates of the AFCS with an accuracy of ±23.5 Mg C ha^−1^. According to this map, the natural forests in the province currently store 265 million Mg C, with a density of 72 Mg C ha^−1^, as aboveground biomass. Using a spatially explicit modeling technique we derived time-series AFCS maps up to the year 2030 under three forest policy scenarios: business as usual, conservation, and concession. The spatial patterns of AFCS and their trends under different scenarios vary on a local scale, and some areas are highlighted that are at eminent risk of carbon emission. Based on the business as usual scenario, the current AFCS could decrease by 75 %, which may lead to the release of 747 million Mg CO_2_. The other two scenarios, conservation and concession, suggest the risk reductions by 11 and 59 %, respectively.

**Conclusion:**

The time-series AFCS maps provide spatially explicit scenarios of changes in AFCS. These data may aid in planning Reducing Emissions from Deforestation and forest Degradation in developing countries projects in the study area, and stimulate the development of AFCS maps for other regions of tropical Asia.

## Background

Across the world, existing tropical forest landscapes are undergoing rapid deforestation due to natural disasters, as well as the increasing demand for agricultural land, wood products, energy, and developmental projects. Currently, global forest areas account for 3.85 billion ha, or 26 % of the Earth’s land surface [1], but this area is decreasing at around 13 million ha per year [2]. As deforestation continues, the Earth becomes more susceptible to potentially negative impacts on ecosystems and the overall climate system due to the associated effects on carbon balance, biodiversity, soil, water regulation, and weather patterns. Currently, emissions caused by deforestation worldwide are considered to be very high, and are likely to continue in this way for the coming decades. Tropical forest regions in particular are major potential sources of carbon emissions [3–7]. Reducing Emissions from Deforestation and forest Degradation in developing countries (REDD+) [8] is one of the key global initiatives that aims to conserve forests and reduce carbon emissions. The goal of REDD+ is to connect investors to forest users and offer an economic portfolio for the retention of forest carbon and the avoidance of deforestation, while also slowing the drivers of land use change. As a result, the initiative contributes indirectly to biodiversity conservation by helping to reduce habitat loss and ensure the continuation of normal ecosystem services; hence, it is considered a sustainable option for the maintenance of forests. Meaningful implementation of REDD+ requires accurate, high-resolution, spatially explicit maps of forested areas and forest carbon stocks, as well as predictions of their change in the future. Therefore, efforts to improve the methods for mapping forest extents and forest-related carbon stocks, as well as identifying their changes, have been advancing in many parts of the world, including tropical Asia [5, 9–13]. Remote sensing and spatial modeling techniques offer a practical means to monitor and examine changes in forest cover, analyze the implications of forest policies, predict spatial patterns of forest cover in the future, and relate these patterns to carbon stock densities [9, 14–16].

Accurate mapping of aboveground forest carbon stocks (AFCS) using spaceborne satellite data is still very challenging due to the requirement of a large amount of in situ data for forest carbon estimation model calibration and validation. Although traditional plot-based field measurements of AFCS have proven most accurate, they are costly and difficult to implement for large areas with dense tropical forests. Studies [13, 14, 17] have shown that light detection and ranging (LiDAR) techniques allow the accurate measurements of geographically referenced vertical forest structures, including canopy height, volume, and biomass. Using LiDAR data, an allometric model for AFCS can be developed with a relatively small number of field measurements [13, 17]. Modeling results can be used to extend the field data, providing spatially extensive and detailed forest attribute data, that can be used to calibrate AFCS predictive models build around a wide variety of spaceborne data, including synthetic aperture radar (SAR) and optical imageries covering larger areas [14, 18].

The integration of both airborne and spaceborne remote sensing techniques has offered the opportunity to more precisely map forest cover and related carbon stocks over wider areas at suitable spatiotemporal scales [6, 13, 14, 19]. However, the potential application of optical spaceborne remote sensing data in Asian tropical forest regions is limited, due to the frequent appearance of clouds and haze, as well as the insensitivity of sensing systems to the variability of biomass with a multi-layer canopy in highly dense forests. In contrast, spaceborne SAR is not limited by these factors as it penetrates clouds to image the Earth’s surface regardless of weather conditions or solar illumination. Among the available spaceborne SAR systems, the Advanced Land Observation Satellite (ALOS) Phase Arrayed L-band SAR (PALSAR) operating at a wavelength of 23.6 cm is very sensitive to forest structure, yielding valuable information for the mapping of forest cover [20–22] and AFCS measurements [23, 24]. However, studies have shown that saturation remains a dominant issue when directly estimating AFCS using SAR data in high biomass areas [25–29]. A consideration of multi-temporal SAR data with multiple polarizations and the use of rule-based algorithms can help to mitigate the saturation problem and improve AFCS estimations [14, 24].

**Fig. 1:**
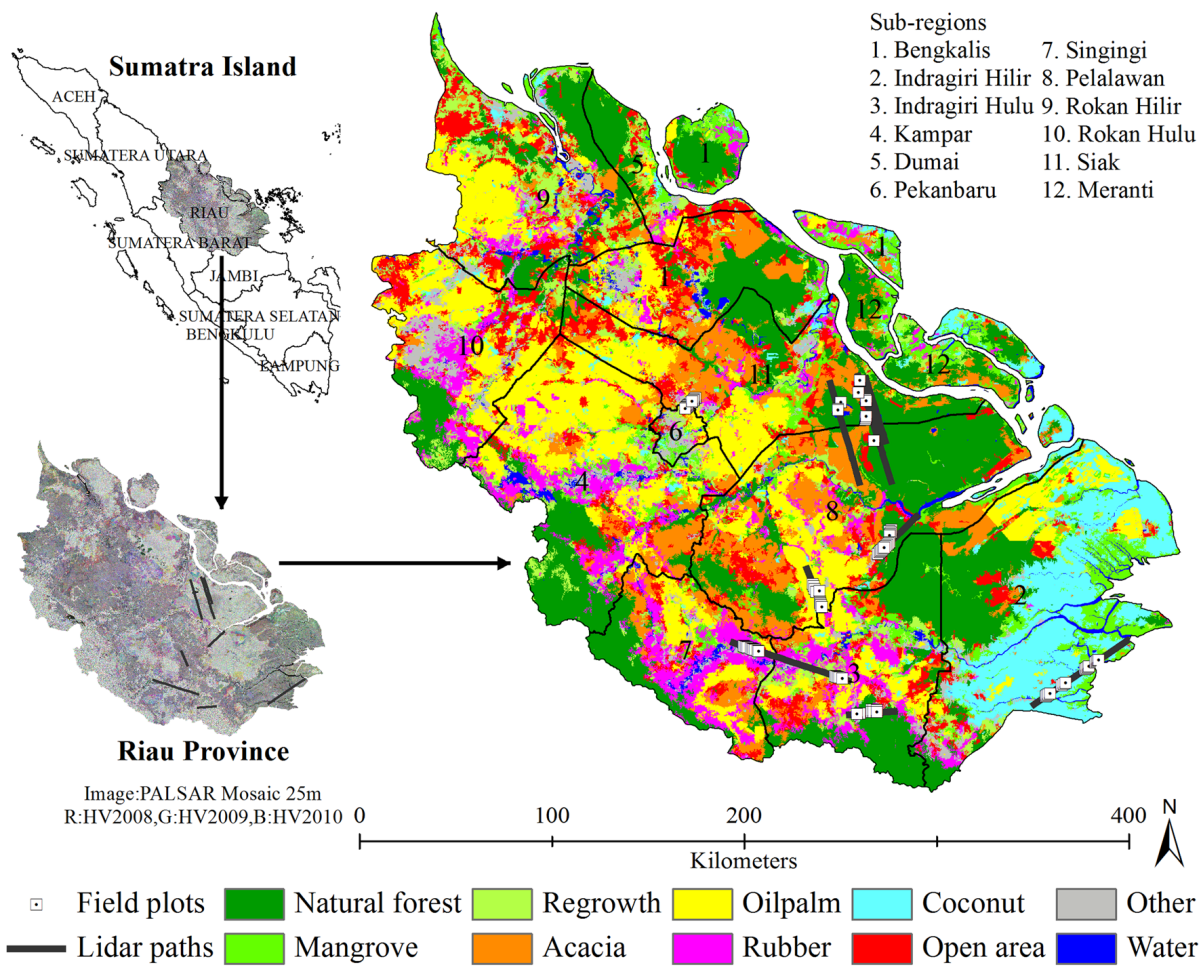
Study area, showing the location, composite PALSAR image, land use and land cover map derived from PALSAR data analysis [35], locations of field measurement plots, and LiDAR data acquisition paths

In addition to remote sensing techniques, spatial modeling is required in order to visualize and quantify the future variations of AFCS [3, 9, 15]. Future trends are reliant on the past processes of deforestation, and represent a consolidation of the relationships between time, space, and driving factors. A logically developed spatial model incorporates these relationships and extrapolates the likelihoods of various forest spatial patterns into the future [15, 16]. Such models offer a means of examining the implications of different forest policies on AFCS, allowing appropriate measures to control deforestation and retain AFCS to be formulated. In this study, our aim is to create a baseline AFCS map of a tropical forest in Asia and to estimate its future AFCS patterns under different forest policy frameworks. The Riau Province in Indonesia (Fig. 1) was chosen as the study site, due to its high carbon emissions as a result of deforestation compared with other provinces in the country [30]. Currently, 5.54 million people live in the province, with an annual growth rate of 3.6 % [16, 31]. Pulp and paper, oil-palm, rubber, and petroleum products are the main sources of income, while the forest landscape has also become a major provider of land in recent years. An ever increasing population and demanding economic activities have increased deforestation and forest degradation, which is ultimately threatening the forest carbon stocks, peat drainage, and biodiversity in the province.

Field measurements, LiDAR data, time-series PALSAR data, and a rule-based algorithm were used together to create a baseline AFCS map with high spatial resolution. The spatial model developed by Thapa et al. [16] was applied to visualize and assess the implications of different forest policies on future AFCS.

## Results and discussion

Table 1 summarizes the field measurement data collected over 87 plots, divided according to the various forest types in the study area. The forests are diverse and exhibit high variability in AFCS of different regions. Around 47 % of the measurement plots are within natural forests, including peat swamps (21.8), dry moist forest (10.3), regrowth (5.7), and mangrove areas (9.2). The remaining plots are within plantation forests including rubber (11.5), acacia (10.3), oil palm (23.0), and coconut (8.0) forests. Such a range of forest types is host to AFCS of 1.18–334.10 Mg C ha^−1^. Among the plots, oil palm was found to have the lowest AFCS, while the natural dry moist forest areas had the highest. The regrowth forest areas had AFCS of 84.58–164.49 Mg C ha^−1^, ranging between that of dry moist forests and peat swamps. Among the plantation forests, rubber plantations had both the highest mean and the maximum AFCS. Across the full range of values, the oil palm plantations had the lowest carbon stock of all the forest types in the study region.

**Table 1:** Summary of field measurement plots and AFCS estimates by forest types

Forest types	Field plots in %	AFCS (Mg C ha^−1^)	
		Mean	Range
*Natural forests*			
Peat swamp	21.8	114.06	69.70–173.62
Dry moist	10.3	198.10	106.69–334.10
Regrowth	5.7	125.15	84.58–164.49
Mangrove	9.2	26.55	9.02–42.99
*Plantation forests*			
Rubber	11.5	38.82	19.10–58.88
Acacia	10.3	32.39	21.04–46.38
Oil palm	23.0	8.27	1.18–20.94
Coconut	8.0	16.00	6.87–27.60

AFCS is 47 % of the field measured aboveground biomass

The maximum likelihood algorithm (MLA) mapping procedure was used to create four maps using the PALSAR gamma-naught image from 2010, three AFCS maps to which the Lee, Frost, and median filters had been applied, respectively, with a 3 × 3 window, and an AFCS map without any filters for comparison. Validation was performed with three individual statistical measures to assess the filtering effects. The median filter provided the best AFCS map, with a RMSE and bias of 27.59 and −0.83 Mg C ha^−1^ and an index of agreement (D) of 0.74. The other two filters, Lee and Frost, gave RMSEs with biases of 28.03 and −1.28, and 27.47 and −3.67 Mg C ha^−1^, and D values of 0.71 and 0.74, respectively. The map created without the application of any filters had an RMSE and bias of 30.09 and −2.71 Mg C ha^−1^, respectively, and a D of 0.59. The D values are similar in both the Frost and the median filters, although the RMSE indicates a slightly better performance with the Frost filtered map, the bias is comparatively high. Overall, these results suggest that the median filter provides a good AFCS mapping product.

**Fig. 2:**
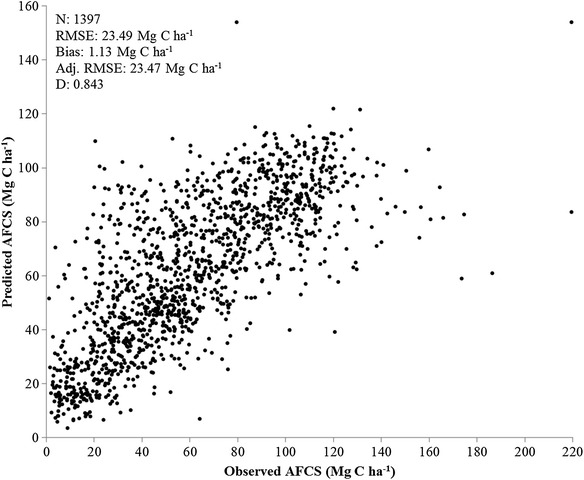
Scatterplot of observed and predicted AFCS values from the validation data set

Four additional AFCS maps were created using the 2010 dataset and four increasing sizes of the filtering window with the median filter. The mapping results gradually improved as the window size increased from 3 × 3 to 9 × 9. Compared with the 3 × 3 window size, the map at the 9 × 9 window size had a reduced RMSE of 25.54 Mg C ha^−1^ with a minimal bias of 0.06 Mg C ha^−1^ and showed high similarity between the predicted and observed AFCS, as indicated by the index of agreement (D = 0.814). However, a decrease in performance was observed with the application of the 11 × 11 window size, with a RMSE and bias of 25.83 and 0.31 Mg C ha^−1^ and a D of 0.812, respectively. This indicated that investigation into larger window sizes is unnecessary; the observed degradation is likely due to overgeneralization of the image data at larger window sizes. As such, a 9 × 9 median filter was applied to PALSAR data from 2009, which was used together with the 2010 data while running the MLA. Inclusion of the 2009 data set improved the AFCS mapping by reducing the mapping uncertainty, and the RMSE and bias dropped to 23.49 and 1.13 Mg C ha^−1^. The value of D also improved to 0.843, indicating a very high similarity between the predicted and observed AFCS values (Fig. 2). Consideration of multi-temporal mosaic data sets can provide better estimates, indicating a normalization of climatic conditions and increasing the potential of replicating the modeling results in other similar tropical regions. It should be noted that after removing the bias in the RMSE, the error was reduced to 23.47 Mg C ha^−1^.

The level of error in this map may have resulted from several unquantifiable factors, including differences in field measurement processes, inaccuracies in the field and LiDAR allometric equations, the slope correction method for the PALSAR mosaic data, and the time difference between field and remote sensing measurements [24]. However, the mapping error produced herein is low, and to date remains unmatched in similar studies of tropical forest regions, such as Kalimantan [27], the Amazon [17], and other areas [19]. Owing to the low mapping error and the high level of similarity between predicted and observed AFCS in such a diverse tropical forest, this map was used as a baseline for a spatial model that calculates future changes in the forest carbon footprint of the study area.

**Fig. 3:**
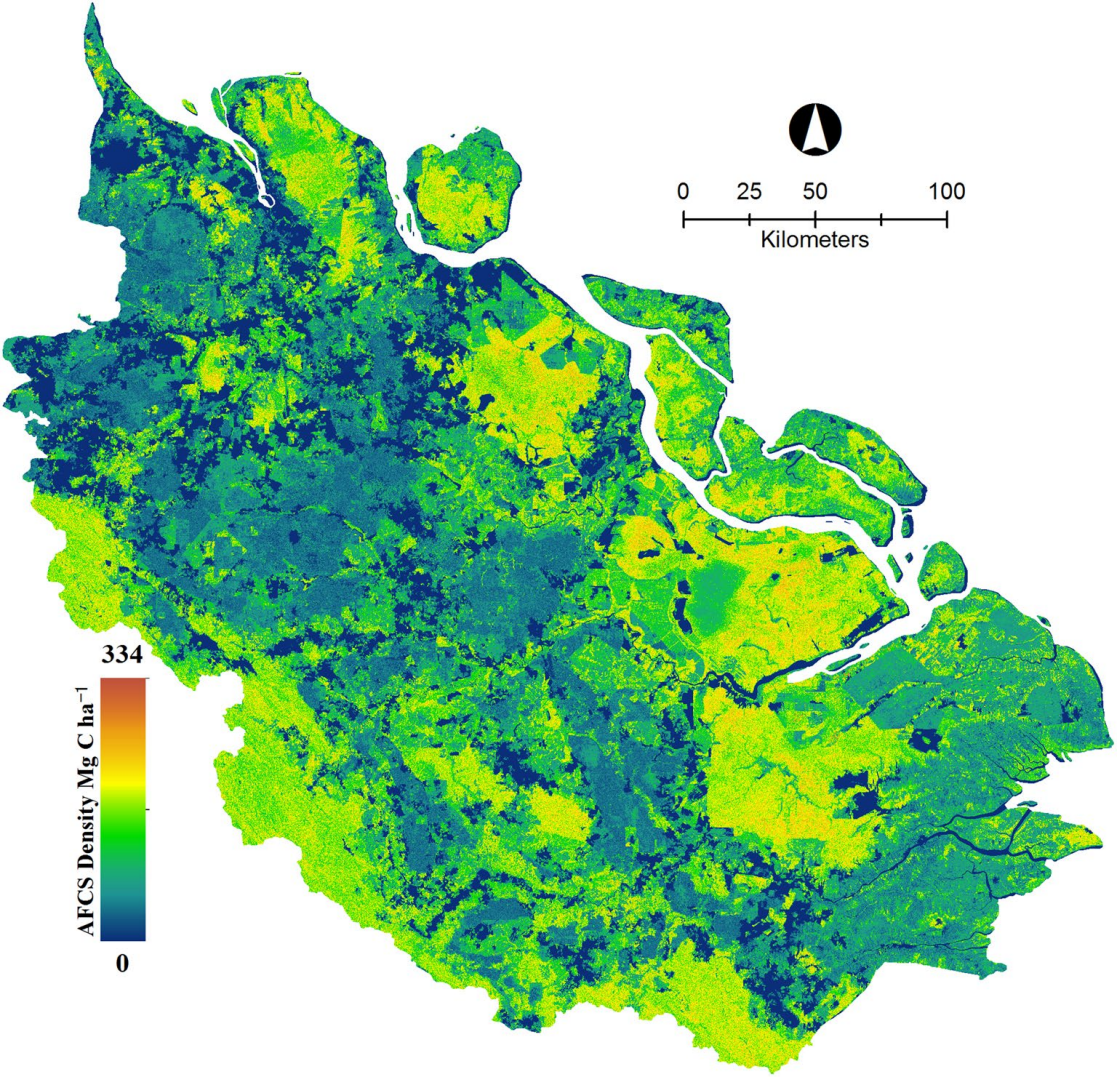
AFCS map, calculated using PALSAR mosaic data

Figure 3 illustrates the baseline AFCS map for the Riau province, in which interesting spatial patterns, with AFCS ranging from <1 to 334 Mg C ha^−1^, can be observed. The spatial variation of AFCS across this map indicates that the majority of areas have carbon densities between 100 and 200 Mg C ha^−1^. High carbon density areas are mostly found within the northern and south-eastern parts of the province, as well as along its western margin. The central area has a generally low carbon density from north to south. These patterns result from the relative distributions of natural forests and plantation forests. The low carbon density areas are mostly covered by plantation forests, agricultural land, and urban forests, while the higher density areas are correlated with existing natural forests, including peat swamps, dry moist forests, and regrowth. The slightly higher AFCS values on the islands in the central-eastern region and on the southeastern margin of the province represent mangrove forests.

The results of this study suggest that the AFCS of the natural forest areas in Riau province, which cover around 3.68 million ha, was 265.57 million Mg at the time of measurement (Table 2). The AFCS density of the natural forest is on average 71.99 Mg C ha^−1^ across the province. Among the different districts, Indragiri Hilir in the southeastern part of the province has the highest AFCS density, at 77.99 Mg C ha^−1^, whereas Pekanbaru in the central region has the lowest density, at 43.63 Mg C ha^−1^. The Pekanbaru district is dominated by urban areas and contains fewer forested areas than the other districts, and therefore has a very little AFCS of 0.33 million Mg. In contrast, the Pelalawan district has the highest AFCS in the province, at 48.79 million Mg, which amounts to 18.37 % of the total AFCS stored in natural forests across the study area. However, the AFCS density in this district is slightly less than the provincial average. In terms of overall AFCS density, the Indragiri Hulu and Meranti districts have a similar quality of forest, as they both have a density of 75 Mg C ha^−1^. Interestingly, the Bengkalis and Siak districts contain a similar amount of AFCS, each with around 10 % of the total carbon stocks, but the AFCS in the Bengkalis districts is slightly higher. Additionally, although the total AFCS of the Dumai district is lower, its density is higher than that in the Singingi district. The Indragiri Hulu district has the second largest AFCS overall, at 33.31 million Mg, and has a density similar to that of the Meranti district. Finally, the Kampar, Rokan Hulu, and Rokan Hilir districts within the northwestern part of the province contain considerable forest carbon stocks, but their densities are slightly lower than the provincial average.

**Table 2:** Quantity of AFCSs extracted for natural forest areas and their distribution by district

Districts	Forest area in ha	AFCSs		
		In million Mg C	% distribution	Density in Mg C ha^−1^
Bengkalis	370,636	26.74	10.07	72.15
Indragiri Hilir	288,914	22.53	8.49	77.99
Indragiri Hulu	443,704	33.31	12.54	75.06
Kampar	433,434	30.50	11.48	70.37
Dumai	101,173	7.22	2.72	71.36
Pekanbaru	7526	0.33	0.12	43.63
Singingi	307,621	20.93	7.88	68.04
Pelalawan	679,426	48.79	18.37	71.81
Rokan Hilir	276,571	18.57	6.99	67.13
Rokan Hulu	217,973	15.64	5.89	71.74
Siak	370,296	26.63	10.03	71.92
Meranti	191,670	14.39	5.42	75.07
Total	3,688,944	265.57	100.00	71.99

Natural forest area is calculated using the Thapa et al. [16] map for 2010

**Fig. 4:**
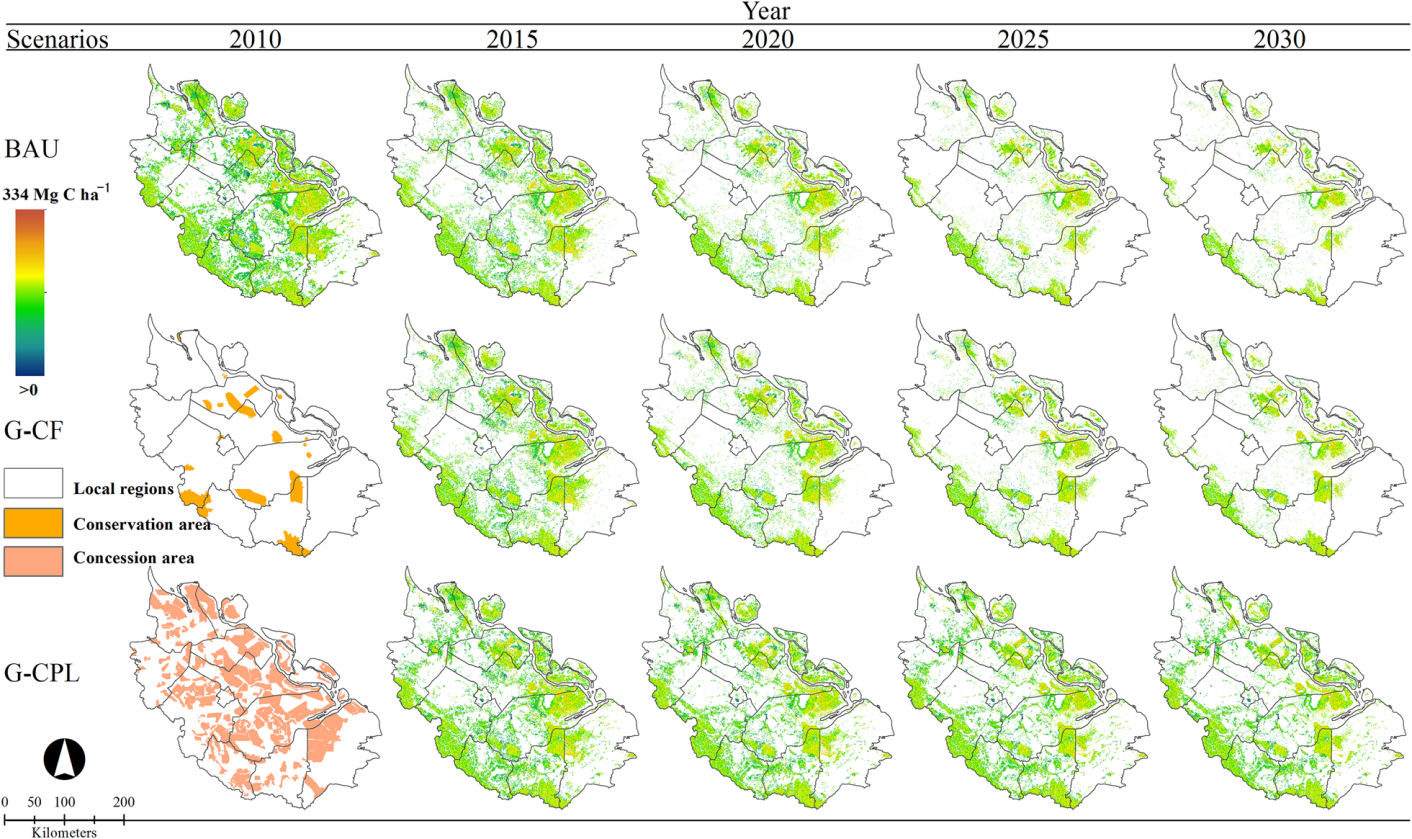
AFCS patterns from 2010 to 2030 under the three forest policy scenarios

We overlaid the AFCS baseline map derived in this study with the observed and simulated scenario-wide forest maps of Thapa et al. [16]. Figure 4 contains information on the distributions of conservation areas, concession areas, as well as illustrating the AFCS map for natural forest cover in 2010, and scenario-wide simulated AFCS maps for the years 2015, 2020, 2025, and 2030. The changes in spatial patterns of AFCS are clear when comparing the two policy scenarios, the Government-Forest Conservation (G-FC) and Government-Concession for Plantation and Logging (G-CPL) policies, with the Business as Usual (BAU) approach. If the past deforestation processes continue without any policy implementation, as shown by the BAU policy scenario, then the AFCS will be persistently released from most of the forested areas. The AFCS removal will likely occur in the most ecologically delicate areas, including the peat swamps and conservation areas in the northeast, and the dry-forested areas in the southwest of the study area. This indicates that the current land use change-inducing activities pose an extremely serious threat to AFCS, and immediate measures are required to ensure sustainability and forest protection. The spatial trends in AFCS observed under the G-CF policy scenario are somewhat comparable to those of the BAU scenario, with the exception of the forest conservation zones. The AFCS hosting areas remain fairly large under the G-CF scenario, due to the impact of the policy on forest protection. For instance, the forests in the designated conservation areas of 2010 remain intact for the forthcoming decades, retaining their AFCS. However, deforestation pressures will still affect the regions outside the conservation areas, rapidly releasing AFCS from the districts in the northern part of the study area through 2015 and 2020. Under the G-CPL scenario, the geographic distribution of AFCS across the province was better retained compared with that of the other scenarios. AFCS removal will likely occur only in the concession lands for plantations and selective logging. If the G-CPL policy is implemented without modification, then entire districts will retain a considerable amounts of their AFCS, even by the end of 2030.

**Fig. 5:**
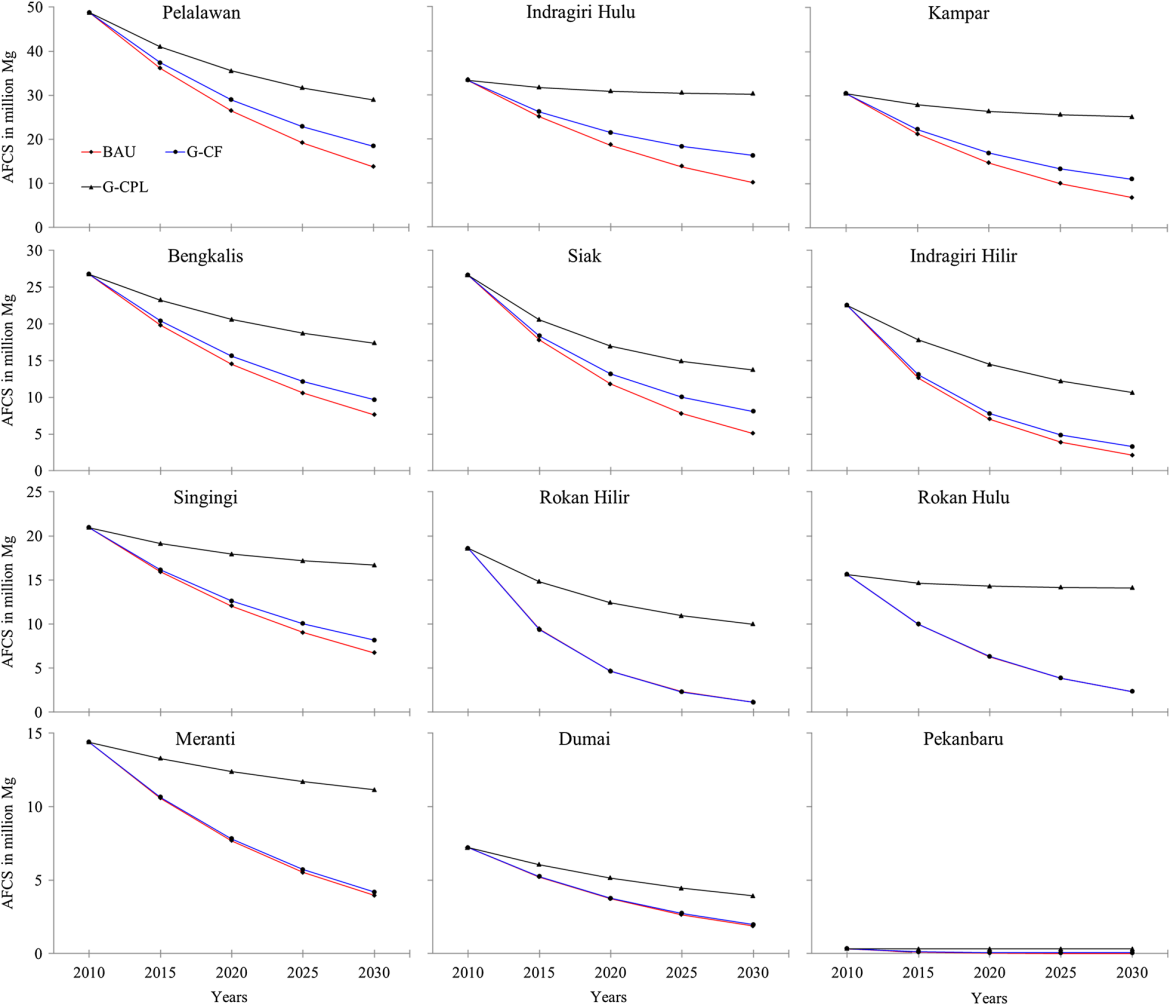
Scenario-specific expected AFCS in each district at 5-year intervals from 2010 to 2030

Figure 5 illustrates quantifications of the predicted AFCS changes under the three policy scenarios by district for every 5 years until 2030. These charts represent important information by showing the spatial variations in future forest carbon reserves. The AFCS curves for each of the different scenarios show the dynamic variability of carbon stocks over time. In all districts and at all times, the BAU line resides at the bottom of the chart, indicating the greatest loss to AFCS. Thus, if the previous tendencies continue, the AFCS in the Pekanbaru district will be almost entirely lost by 2030. In addition, the Dumai, Meranti, Rokan Hulu, and Rokan Hilir districts are likely to face severe damage to their AFCS balances over the next two decades. These districts will also face similar problems under the conservation scenario. The AFCS emissions in the Pelalawan district under the BAU expected to be high, due to the large number of reserves in this region compared with the other districts. The most rapid declines in AFCS up to 2015 under BAU are expected in the Siak, Indragiri Hilir, and Rokan Hilir districts. Over the study period, the changes in projected AFCS between the G-CF and BAU scenarios gradually widen in the Kampar, Pelalawan, Siak, and Indragiri Hulu districts. This indicates that the G-CF scenario somehow retains the stability of forest carbon reserves to a greater degree than the BAU scenario. In comparison, the G-CPL policy scenario estimates the retention of relatively high AFCS in the Meranti, Singini, Indragiri Hulu, and Kampar districts, even by 2030. Remarkably, the estimated AFCS emissions in Indragiri Hulu, Kampar, Rokan Hulu, and Singingi districts under the G-CPL policy scenario are very low over the study period. In contrast, rapidly declining levels of AFCS are detected in the Siak, Dumai, and Indragiri Hilir districts under the G-CPL policy scenario. The remaining districts, including Indragiri Hulu, Rokan Hulu, Meranti, and Singingi, will contain higher AFCS by 2030 in the G-CPL scenario than under the other scenarios.

**Fig. 6:**
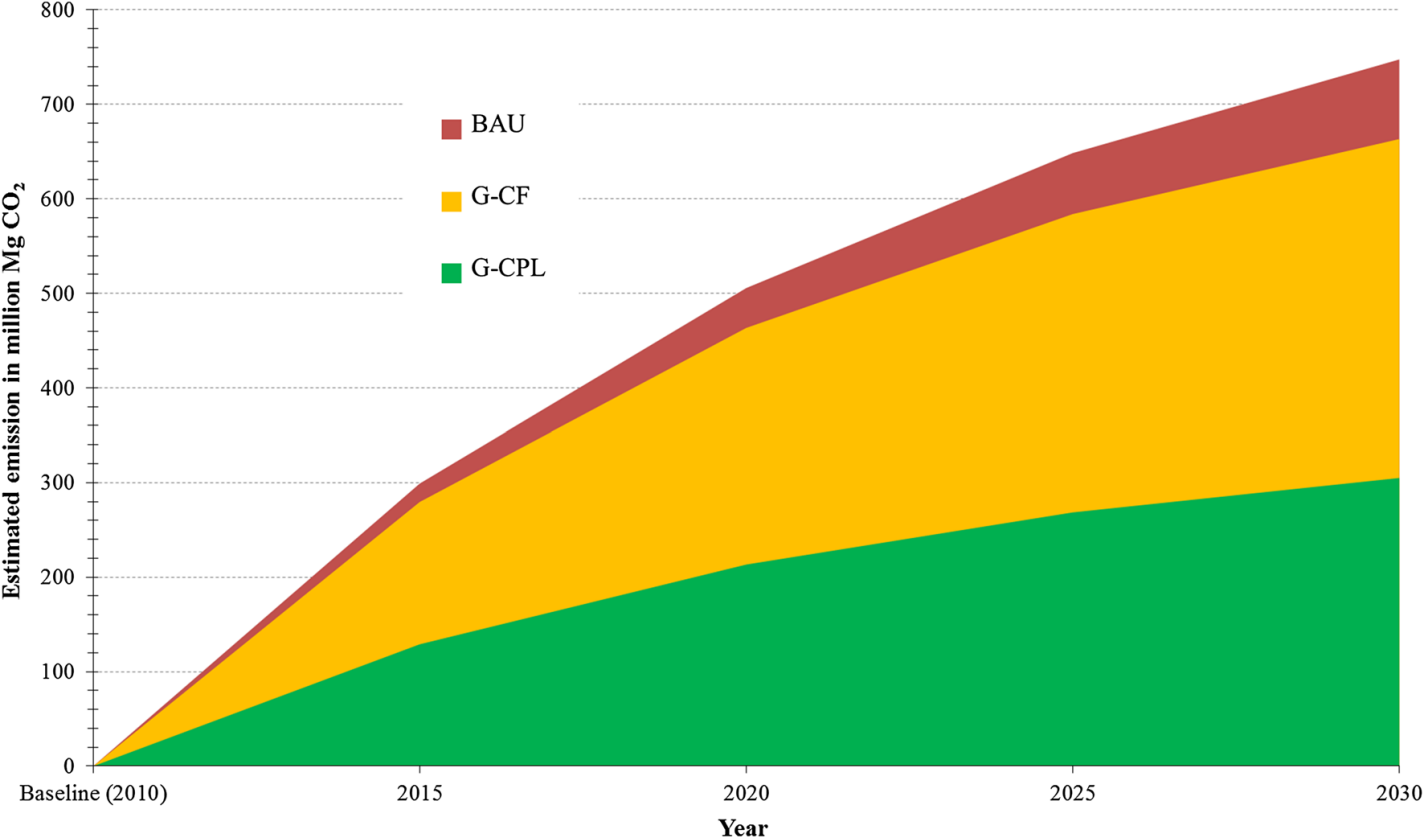
Scenario-specific estimated CO_2_ emissions as a result of deforestation across the province from 2010 to 2030

Figure 6 presents the expected forest carbon emissions from the province as a whole under each scenario in five-year intervals up to 2030. The effects of the different scenarios on the projected carbon emission differ with the passing time. If the existing trend continues, as evidenced by the BAU, around 747.61 million Mg CO_2_, representing 75 % of the current AFCS, will likely be in the atmosphere by 2030. The trend indicates that the emissions will be greater in earlier years, meaning that two thirds of the forest carbon will be emitted into the air over the next 10 years, potentially resulting in globally adverse environmental consequences. In comparison, the trend of the G-CF scenario suggests some measure of success in the form of reduction in emissions of 20 million Mg CO_2_ by 2015, although these double by 2020, and reach approximately 84.3 million Mg CO_2_, a reduction of 11.27 % as compared to the BAU in 2030. In contrast, the emissions under the G-CPL scenario appear to differ remarkably from those under the G-CF and the BAU scenarios. The implementation of this policy will gradually slow the emission of forest carbon stocks by controlling the deforestation to a greater degree than the other scenarios. Thus, under the G-CPL scenario, the estimated carbon emissions will be around 305 million Mg CO_2_ in 2030, which is only 31 % of the current AFCS; this represents a reduction of 2.5 times compared with the BAU scenario. It is worth noting that the G-CPL scenario is likely to delay the carbon emissions by a further 15 years, whereas a similar amount is expected to be released by 2015 under the present conditions. If BAU is considered as a reference scenario, then the concession policy (G-CPL) scenario is likely to reduce the CO_2_ emissions by 59.20 % (442.6 million Mg CO_2_) through its expected deforestation by the end of 2030.

Despite the high spatial resolution of our AFCS map, which reveals the AFCS dynamics of natural forests on a local scale, information about the dynamics of below-ground carbon stocks (BGCS) in natural forest areas is lacking in this study. Estimation of BGCS in natural forest regions using remote sensing is extremely difficult. However, the general model [32, 33] for estimating the BGCS in tropical regions can be employed using the baseline map derived in this study if necessary. In addition to affecting the BGCS, the deforestation process also contributes to the release of carbon from other sources such as soils and peat lands. These additional sources may significantly increase the net carbon emissions, and the inclusion of forest fire parameters in the modeling process may improve the accuracy of future estimation. Furthermore, the majority of the natural forest land in this area has already undergone transformations to produce economically valuable industrial plantations, such as oil palm, acacia, coconut, and rubber trees. These plantation forests also store a significant amount of aboveground forest carbon, as reflected in baseline map (Fig. 3) and in Table 1. From a carbon stock perspective, a consideration of the AFCS dynamics in these forests may represent a trade off in overall carbon balance to some extent. However, there is an immediate risk of carbon emission when the natural forests are cleared, even when they are replaced by plantation forests. Additionally, these plantation forests store carbon only in the short term, as they are harvested within a certain period of time. For example, an acacia plantation in the study area is harvested every 4 years. The other plantations, such as oil palm, coconut, and rubber are often harvested every 20–30 years. The plantation forests maintain greenness in the province but still have a significant impact on carbon recycling and prevent the restoration of ecosystem services.

## Conclusion

Through the integration of multiple remote sensing techniques, from airborne LiDAR to spaceborne SAR with field measurement data and a rule-based algorithm, an accurate baseline AFCS map with high spatial resolution was developed for one of the major tropical forests in Asia. This baseline map provides highly accurate, spatially explicit distributions and quantitative estimations of forest carbon stocks in the study area. The AFCS distribution varies geographically, indicating spatial variations in forest quality and vulnerability. The spatial modeling technique provides an opportunity to extrapolate the spatial trends in AFCS and examine the implications of different forest management policies on carbon stocks and emissions over the next two decades. The inherent capability of the model to distinguish local variations in future AFCS trends under different scenarios is key to identifying the areas most vulnerable to high carbon emissions, which would require immediate mitigation measures to ensure forest conservation. The model was used to predict the spatiotemporal variations and associated quantities of remaining AFCS under different scenarios up until the year 2030. These predicted spatial patterns of AFCS indicate that the forest carbon emission rate is likely to be high in the coming decades across the province. Ongoing deforestation is expected to release around 747 million Mg CO_2_ into the atmosphere by 2030. A forest conservation policy will slow the AFCS emissions, but the reduction will be insufficient. Among the scenarios tested, the concession scenario is the most promising, halving the expected emissions if it is implemented as planned. In addition to the high-resolution AFCS map, the modeling outcomes may provide opportunities for the identification of especially vulnerable localities and focuses for the implementation of REDD+ projects to obtain the greatest benefits based on the environmental settings. For the proper execution of the REDD+ project, it is important to understand how the expected trends in AFCS distribution are likely be affected in the long term by the implementation various plans, policies, and strategies. The AFCS under the BAU scenario may provide a reference emission scenario for REDD+, while the other scenarios can be used as examples in the initial exploration of the range of potential spatiotemporal issues and outcomes. These can provide important insights for preparedness activities that mitigate the problem of forest carbon emissions. The spatially explicit AFCS map and the modeled scenario results will therefore contribute to the sustainable management of forests in the study area and to the formulation of REDD+ projects, as well as representing a methodological reference for wider audiences in tropical regions and beyond.

## Methods

### L-band SAR data collection and processing

The study area spans more than 9 million ha commonly experiences cloudy and hazy skies throughout the year [20]. This degradation in atmospheric conditions over the study area precludes use of optical remote sensing techniques in assessing forest quality and AFCS. As a result, PALSAR mosaic data used for tropical forest monitoring as they are unaffected by atmospheric condition and are available for wall-to-wall mapping [1, 20]. The mosaic data are slope-corrected and orthorectified using the widely available SRTM 90 m digital elevation model without any alteration the image quality [21]. Currently, 25 m global mosaic data in two polarizations (HH and HV) are available as one set per year, from 2007 to 2010. The mosaic data are available at a downloadable size of 1-degree tiles, equivalent to approximately 111 × 111 km [34]. Those mosaic products covering the whole province of Riau for the years 2009 and 2010 were used. The mosaic data were converted into radar backscatter coefficients using gamma-naught (γ°) [21] due to high sensitivity to forest structure and its usefulness in forest cover analysis [20, 35].

To improve the confidence of the AFCS mapping, we also examined whether a particular filter or its size would affect the mapping results. Three filters were examined: the Lee, Frost, and median filters. These filters possess different formulations and assumptions for smooth speckled data in radar imagery. The Lee filter is a standard deviation-based filter, and filters data on the basis of statistics calculated within individual filtering windows. It conserves image sharpness and details while reducing speckle noise. The value of the pixel being filtered is replaced by a value computed using the neighboring pixels. In comparison, the Frost filter is an exponentially damped circularly symmetric filter, which utilizes local statistics. The value of the pixel being filtered is replaced by a value computed with a consideration of the damping factor, the local variance, and the distance from the filter center. This filter is able to preserve the edges in the images. The median filter reduces the speckle noise in an image by conserving edges greater than the kernel dimensions. It replaces the value of each center pixel with the median value of the neighborhood specified by the filter size. In order to investigate the impact of speckle filtering sizes on the mapping, we also evaluated five different filtering window sizes (3 × 3, 5 × 5, 7 × 7, 9 × 9, and 11 × 11).

### Field data collection and processing

The combination of LiDAR data and plot-based field measurements has emerged as a promising technique for accurately estimating AFCS [13, 17]. We conducted field measurements and airborne LiDAR surveys within the province during 2012 and 2013. Owing to the differences in forest structure and associated biomass in different land use and land cover (LULC) types, we adopted a stratified sampling approach based on the major forest types to determine the locations (Fig. 1) for field and LiDAR measurements. The major forest types in the study site were defined as natural forests, including peat swamps, dry moist, mangrove, and regrowth, and plantations including acacia, oil palm, rubber, and coconut. Based on these forest types, eight strata were created.

Across the field measurement campaigns, we made 87 biomass measurements within 1 ha-size plots that coincided with the LiDAR acquisition sites. Forest stands of all ages were inventoried, from mature to recent regrowth. Owing to the time and cost involved in conducting a census-based measurement of all trees in a 1-ha-sized plot, a sub-sampling approach was adopted using representative subplots. The sub-sampling methods differed between the natural and plantation forests.

To determine woody biomass, all living and standing deadwood trees with a diameter at breast height (at 1.3 m; DBH) ≥5 cm were measured in each subplot. We used allometric equations previously developed for the specific forest types: peat swamp forest [36], dry moist forest [37], mangrove [38], acacia [39], rubber [40], coconut and non-trees [41], oil palm [42], standing deadwood [43], lying deadwood [44], and bamboo [45]. The biomass of understory vegetation and litter was calculated by multiplying the mass of a fresh sample measured in the field by the ratio of sub-sample dry mass to sub-sample fresh mass. The plots within regrowth forests were all located in either peat swamps or dry moist forested areas. Therefore, the biomass for this class was calculated using the corresponding allometric equation, based on its location. Detailed descriptions of the field measurement method, plot specification, and allometric equations used to convert the field measured data to plot level aboveground biomass (AGB) are presented in Thapa et al. [13, 24]. In this study, the AFCS for each plot was considered to be 47 % of the field measured AGB [46].

Airborne LiDAR surveys were conducted at eight sites. LiDAR data were acquired on fine weather days in February 2012 and during November–December 2012. In February, we used an LM-5600 laser system operated at approximately 1000 m above the ground. This system captured first and last returns at a scan angle of ±20°. The average point density was 1.2 m^−2^. During this measurement period, three of the eight sites were surveyed, covering 3600 ha of forested land. The remaining LiDAR data were collected using an Optech ALTM 3100EA laser system in the second survey period. This system was operated approximately 600 m above the ground, and captured full waveform data at a scan angle of ±32°. Discrete return data were recorded at average point density of 3.6 m^−2^. At this time, 4472 ha of forested lands were surveyed, contributing to a total 8072 ha of LiDAR data acquired during the two periods combined. Further details of the LiDAR systems, data acquisition and processing, and the LiDAR-to-AFCS model calibration and validation are discussed in Thapa et al. [13]. In the present study, the LiDAR allometric model (Eq. 1) was used to create additional AFCS plots as the AFCS model was calibrated and validated over the same area [13].

1 $$\begin{aligned} {\text{AFCS}}\,\,{\text{Mg}}\,\,{\text{C}}\,\,{\text{ha}}^{ - 1} & = 259.488 - \left( {146.373 \times {\text{MCH}}} \right) + \left( {4.738 \times {\text{MCH}}^{2} } \right) - \left( {4.881 \times {\text{Cover}}} \right) \\ & \quad + \left( {3.513 \times {\text{MCH}}\_{\text{cover}}} \right) - \left( {0.0954 \times {\text{MCH}}^{2} \_{\text{cover}}} \right) - \left( {1.583 \times {\text{QMCH}}\_{\text{cover}}} \right) \\ & \quad + \left( {22.568 \times {\text{P}}50} \right) + \left( {26.118 \times P90} \right) \\ \end{aligned}$$

where, MCH = mean canopy height, Cover = forest cover as a percentage of all returns above the MCH, MCH_cover = MCH × Cover, MCH^2^_cover = MCH^2^ × Cover, QMCH_cover = quadratic MCH × Cover, and P50 and P90 are the 50th and 90th percentiles of canopy height, respectively.

A total of 2716 1-ha LiDAR-based AFCS plots were created, avoiding an excess of path boundaries, field measurement plots, agricultural fields, clear cut areas, water areas, and built structures including buildings and roads. These LiDAR AFCS plots were combined with the 87 field measurement plots, resulting in a total of 2803 plots available for calibration and validation of the SAR-based AFCS baseline map.

### Mapping and validating the AFCS

A supervised rule-based approach was adopted for the AFCS mapping, as the direct relationship between the PALSAR backscattered coefficients and field-measured AFCS in the study region is affected by saturation in higher biomass areas [24]. This approach analyzes the spatial patterns in the PALSAR mosaic data through the use of training samples and the rules formulated in a maximum likelihood algorithm (MLA) [47], as in Eq. 2, to determine the AFCS value for each PALSAR pixel. Half of the total AFCS plots were used to train the algorithm. The MLA quantitatively examines both the variance and the covariance of the patterns of corresponding backscatter in the training samples and provides the most probable AFCS values for the selected pixels. At first, the MLA was applied to the 2010 gamma naught image, which includes HH and HV polarizations. The different impacts of the three filters (Lee, Frost, and median) and five increasing window sizes on the mapping results were analyzed. Then, we investigated whether the addition of the gamma naught image of the previous year, in the form of mosaic data from 2009, would improve the mapping result. Layer stacking was performed while adding the 2009 image layers (HH and HV) to the 2010 image data set.

2 $$g_{i} (x) = \ln p(\omega_{i} ) - \frac{1}{2}\ln \left |\mathop \sum \limits_{i} \right | - \frac{1}{2}(x - m_{i} )^{t} \mathop \sum \limits_{i}^{ - 1} (x - m_{i} )$$

where *g*_*i*_ is the AFCS Mg C ha^−1^ corresponding to the training sample, in which *i* = 1…C (the number of AFCS training plots available); *x* is the position, in n-dimensional data where n is the number of image data layers (for example, 2 per year); *p*(*ω*_*i*_) is the probability that the AFCS value *ω*_*i*_ occurs in the image; |Σ_i_| is the covariance matrix of the image data intersected with the spatial size of AFCS plot *ω*_*i*_; *t* is the transposition of the matrix; Σ_i_^−1^ is the inverse matrix; and m_i_ is the mean backscatter vector corresponding to AFCS plot *ω*_*i*_.

A validation map was prepared using the remaining half of the sample plots. The AFCS map was compared with the validation map through the calculation of three statistical mapping uncertainty measures: root mean square error (RMSE, Eq. 3), bias (Eq. 4), and index of agreement (D, Eq. 5).

3 $${\text{RMSE}}\,\,({\text{Mg}}\,\,{\text C}\,\,{\text{ha}}^{ - 1} ) = \sqrt {\frac{{\mathop \sum \nolimits_{i = 1}^{n} (P_{i} - O_{i} )^{2} }}{n}}$$

4 $${\text{Bias}}\,\,({\text{Mg}}\,\,{\text C}\,\,{\text{ha}}^{ - 1} ) = \frac{{\mathop \sum \nolimits_{i = 1}^{n} (P_{i} - O_{i} )}}{n}$$

5 $$D = 1 - \frac{{\mathop \sum \nolimits_{i = 1}^{n} (P_{i} - O_{i} )^{2} }}{{\mathop \sum \nolimits_{i = 1}^{n} (|P_{i} - \bar{O}| + |O_{i} - \bar{O}|)^{2} }}$$

where *P*_*i*_ represents predicted values, *O*_*i*_ represents observed values, $\bar{O}$ is the observed mean, and n is the number of observations. D is the index of agreement proposed by Willmott and Wicks [48]. D ranges from 0 to 1, which corresponds to disagreement or perfect agreement between the predicted and observed values, respectively.

Bias-adjusted RMSE (Eq. 6) was calculated for the final map to ensure the high accuracy of the AFCS estimation in the map.

6 $${\text{Adj}} .\,\, {\text{RMSE}}\,\,({\text{Mg}}\,\,{\text C}\,\,{\text{ha}}^{ - 1} ) = \sqrt {\frac{{\mathop \sum \nolimits_{i = 1}^{n} (P_{i} - O_{i} - Bias)^{2} }}{n}}$$

### Mapping of future expected AFCS footprints

In this study, we used the forest cover map derived from PALSAR mosaic data from 2010, in combination with the scenario maps for 2015, 2020, 2025, and 2030 from Thapa et al. [16] to generate the expected AFCS footprints for the various years in the future. Three policy scenarios were analyzed: BAU, corresponding to the ‘business as usual policy’, G-FC indicating the ‘government-forest conservation policy’, and G-CPL, representing the ‘government-concession for plantations and logging policy’. The BAU policy scenario assumes that the deforestation process will continue with the same past trend everywhere in the province, and therefore, AFCS removal will occur in the corresponding deforested areas. The G-FC policy scenario assumes that the deforestation process does not follow the past trend and, in the future, will likely occur outside the designated forest conservation areas. In this case, the forest carbon stocks remain untouched within the conservation areas. For the G-CPL policy scenario, we assume that the concession areas are allotted for selective logging and industrial plantations, and imminent deforestation likely occurs only in the concession areas, therefore the AFCS will be untouched outside these areas. Scenario-wide AFCS maps were created at five-year intervals from 2015 to 2030. Using these maps, the AFCS was quantified at the district level to identify local variation in the carbon stocks. Furthermore, the expected CO_2_ emissions for each scenario were computed at province level for each time interval using the approach of IPCC [46].
